# Bioluminescence imaging of chronic *Trypanosoma cruzi* infections reveals tissue-specific parasite dynamics and heart disease in the absence of locally persistent infection

**DOI:** 10.1111/cmi.12297

**Published:** 2014-05-01

**Authors:** Michael D Lewis, Amanda Fortes Francisco, Martin C Taylor, Hollie Burrell-Saward, Alex P McLatchie, Michael A Miles, John M Kelly

**Affiliations:** 1 Department of Pathogen Molecular Biology, Faculty of Infectious and Tropical Diseases, London School of Hygiene and Tropical MedicineKeppel Street, London, WC1E 7HT, UK; 2 Department of Immunology and Infection, Faculty of Infectious and Tropical Diseases, London School of Hygiene and Tropical MedicineKeppel Street, London, WC1E 7HT, UK

## Abstract

**Summary:**

Chronic *Trypanosoma cruzi* infections lead to cardiomyopathy in 20–30% of cases. A causal link between cardiac infection and pathology has been difficult to establish because of a lack of robust methods to detect scarce, focally distributed parasites within tissues. We developed a highly sensitive bioluminescence imaging system based on *T. cruzi* expressing a novel luciferase that emits tissue-penetrating orange-red light. This enabled long-term serial evaluation of parasite burdens in individual mice with an *in vivo* limit of detection of significantly less than 1000 parasites. Parasite distributions during chronic infections were highly focal and spatiotemporally dynamic, but did not localize to the heart. End-point *ex vivo* bioluminescence imaging allowed tissue-specific quantification of parasite loads with minimal sampling bias. During chronic infections, the gastro-intestinal tract, specifically the colon and stomach, was the only site where *T. cruzi* infection was consistently observed. Quantitative PCR-inferred parasite loads correlated with *ex vivo* bioluminescence and confirmed the gut as the parasite reservoir. Chronically infected mice developed myocarditis and cardiac fibrosis, despite the absence of locally persistent parasites. These data identify the gut as a permissive niche for long-term *T. cruzi* infection and show that canonical features of Chagas disease can occur without continual myocardium-specific infection.

## Introduction

A wide variety of pathogens establish persistent infections by continually evading host immune-mediated clearance and often cause chronic debilitating or fatal illnesses as a consequence. The strategies that microbes employ to ensure their long-term survival within mammalian hosts include antigenic variation, establishment of sites of latency and direct immunomodulation. The protozoan parasite *Trypanosoma cruzi* is an important zoonotic pathogen which establishes life-long infection in humans and causes Chagas disease (American trypanosomiasis). While the cell invasion and intracellular survival mechanisms adopted by *T. cruzi* in acute infections are becoming clearer, the adaptations that allow it to sustain chronic infections remain poorly understood (Nagajyothi *et al*., 2012).

At least 8 million people in Latin America are estimated to be infected with *T. cruzi*, resulting in ∼ 13 000 deaths annually and a significant morbidity burden (Moncayo and Silveira, 2009). Migration patterns are also driving globalization of the disease. For example, recent estimates indicate there are 300 000 infected people living in the USA (Bern and Montgomery, 2009) and up to 123 000 in Europe (Basile *et al*., 2011). Transmission is primarily mediated by triatomine bug vectors but the congenital route, consumption of contaminated food and drink, organ transplantation and blood transfusion are all important additional infection risks (Marin-Neto *et al*., 2008).

Clinical forms of human Chagas disease are divided into distinct stages (Rassi *et al*., 2010). The acute phase is often asymptomatic but can be severe, particularly in children, with death occurring in up to 5% of diagnosed cases. Although sterile immunity is not achieved, most infected individuals suppress parasitaemia within 4–8 weeks and enter the ‘indeterminate’ asymptomatic phase of chronic infection. Clinical manifestations generally do not then occur until decades later. These include chronic chagasic cardiomyopathy (CCC) in 20–30% of those infected, and/or digestive megasyndromes in ∼ 10% of cases (Köberle, 1970; Arantes *et al*., 2004). Factors that influence progression from asymptomatic to symptomatic cardiac disease states are poorly understood and the mechanism(s) of CCC pathogenesis has been controversial (Gironès and Fresno, 2003; Tarleton, 2003; Marin-Neto *et al*., 2007; Bonney and Engman, 2008; Dutra and Gollob, 2008; Gutierrez *et al*., 2009). Damage to the myocardium has been attributed to injury mediated directly by the parasite, by parasite-specific inflammatory responses, and auto-reactive immune responses. The scarcity of parasites in histological sections from CCC patients and the presence of auto-reactive antibodies and T-lymphocytes in chronically infected hosts (Cunha-Neto *et al*., 2011) has led many to favour autoimmune-mediated damage to the heart as the central, or only, pathologically relevant process (Teixeira *et al*., 2011). Strong evidence now supports the continued presence of *T. cruzi* in the host as a requirement for disease development. Molecular methods have confirmed the presence of parasite DNA and antigens in damaged tissues (Higuchi *et al*., 1993; Jones *et al*., 1993; Añez *et al*., 1999), and improved clinical outcomes have been observed in some, but not all, trials of anti-parasitic chemotherapy (reviewed in Marin-Neto *et al*., 2008). A consensus has developed that disease pathology results mainly from pro-inflammatory immune responses that are driven by the continued presence of the parasite in the host. Within this framework, however, the relative contributions of parasite-specific and autoimmune responses to myocardial damage remain unclear. The factors that allow *T. cruzi* to continually evade immune clearance, despite a vigorous adaptive response (Padilla *et al*., 2009), are also unknown. Furthermore, the reason(s) why heart tissue is a major site of disease pathology are not adequately explained by existing data. Evidence for a quantitative association between disease progression and cardiac parasite burden during the chronic stage is equivocal, and the cellular and molecular processes underlying the development of myocarditis and fibrosis are poorly defined.

Mouse models of chronic Chagas disease have fundamental drawbacks that have constrained their use in studying pathogenesis. Unlike the acute phase, where non-dividing trypomastigotes can be detected in blood by microscopy, parasitaemia in the chronic phase is generally subpatent. In these long-term infections, most parasites are intracellular replicative amastigotes, and existing evidence indicates that they reside predominantly in muscle tissues (Zhang and Tarleton, 1999). Adipocytes have been suggested as a possible secondary reservoir site (Matos Ferreira *et al*., 2011; Nagajyothi *et al*., 2012). End-point assays such as histology, PCR and serology require large animal cohorts and the utility of such data is typically limited by tissue sampling biases and the focal nature of tissue infections. Bioluminescence imaging approaches have been developed but have not been applied beyond the acute phase (Hyland *et al*., 2008; Canavaci *et al*., 2010; Andriani *et al*., 2011).

We have developed an enhanced *in vivo* imaging system based on *T. cruzi* expressing a novel firefly luciferase variant that emits tissue-penetrating orange-red light. This enabled highly sensitive *in vivo* and *ex vivo* imaging and long-term, serial evaluation of infections in individual mice. This has allowed us to investigate the association between tissue tropism and development of chagasic heart disease with minimal tissue sampling bias. We found that chronic infections are quantitatively and spatially dynamic and identified the gastrointestinal tract (GIT) as the primary site of parasite persistence. Importantly, *T. cruzi*-infected mice developed chronic myocarditis and cardiac fibrosis, despite the absence of detectable parasites in the heart tissue itself.

## Results

### Highly sensitive *in vivo* imaging of *T. cruzi* expressing red-shifted luciferase

**Figure 1:**
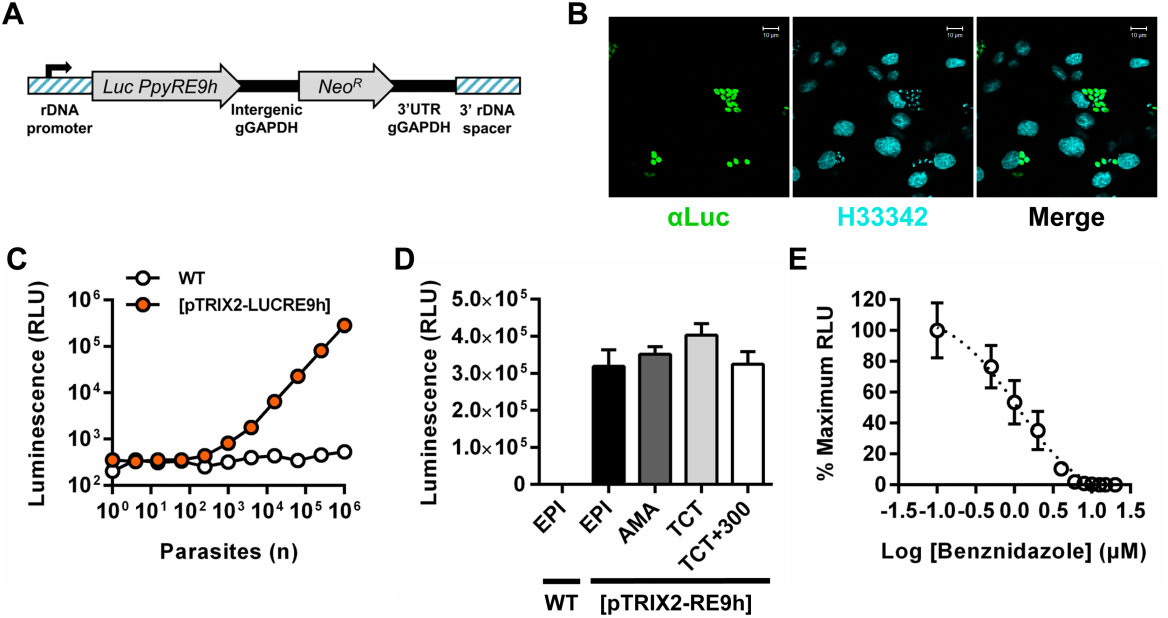
Generation of a *T. cruzi* CL Brener cell line that constitutively expresses *PpyRE9h* red-shifted luciferase.
DNA construct pTRIX2-RE9h, which targets *PpyRE9h* to the rDNA locus.Paraformaldehyde fixed L6 rat myoblasts containing intracellular bioluminescent amastigotes stained with anti-luciferase IgG (αLuc, green) and Hoechst 33342 DNA stain (H33342, cyan).*In vitro* luciferase activity assay of lysates from serially diluted wild-type (WT) and transgenic ([pTRIX2-LUCRE9h]) tissue culture trypomastigotes.Luciferase activity assays of lysates of 1 × 10^6^ wild-type (WT) and transgenic ([pTRIX2-LUCRE9h]) epimastigotes (EPI) and transgenic amastigotes (AMA), tissue culture trypomastigotes (TCT) and TCTs after *in vivo* passages and *ex vivo* culture equating to > 300 days in the absence of G418 drug selection (TCT+300). Data are means + SEM of three to four biological replicates.Luciferase activity assay of lysates from L6 rat myoblast monolayers infected with bioluminescent amastigotes and treated with increasing concentrations of benznidazole; data are mean ± SD of triplicate wells.

We previously demonstrated that the thermostable red-shifted firefly luciferase *PpyRE9h* (Branchini *et al*., 2010) can be used as a highly sensitive reporter for *in vivo* imaging of experimental African trypanosome infections, with dramatically improved bioluminescence detection compared to wild-type firefly luciferase (McLatchie *et al*., 2013). We therefore integrated *PpyRE9h* into the rDNA locus of *T. cruzi* CL Brener using construct pTRIX2-RE9h (*Experimental procedures*; Fig. 1A and Fig. S1). G418-resistant parasites were screened for luciferase activity and a highly expressing clone was selected for *in vivo* experiments. When cultured as epimastigotes, this clone grew at the same rate as wild-type parasites and differentiated into metacyclic trypomastigotes that infected L6 myoblasts normally. Staining with anti-firefly luciferase polyclonal antibody showed that expression was cytosolic, with low intra-population variation in intracellular amastigotes (Fig. 1B). There was a linear relationship between parasite number and luciferase activity *in vitro* (10^3^–10^6^ cells range, *R*^2^ = 0.999) (Fig. 1C). Luciferase expression was similar in epimastigotes, amastigotes, and tissue culture trypomastigotes (Fig. 1D). Long-term constitutive luciferase expression was preserved as shown by activity assays performed on parasites maintained for > 300 days in the absence of selective drug-pressure (Fig. 1D), using a regimen that included passage through a severe combined immunodeficient (SCID) mouse, inoculation into a BALB/c mouse and recovery by *ex vivo* culture at 170 days post-infection (dpi). Benznidazole treatment of *in vitro* infected L6 myoblasts harbouring amastigotes resulted in a dose-dependent reduction in luciferase activity with an IC_50_ of 1.07 μM (Fig. 1E).

**Figure 2:**
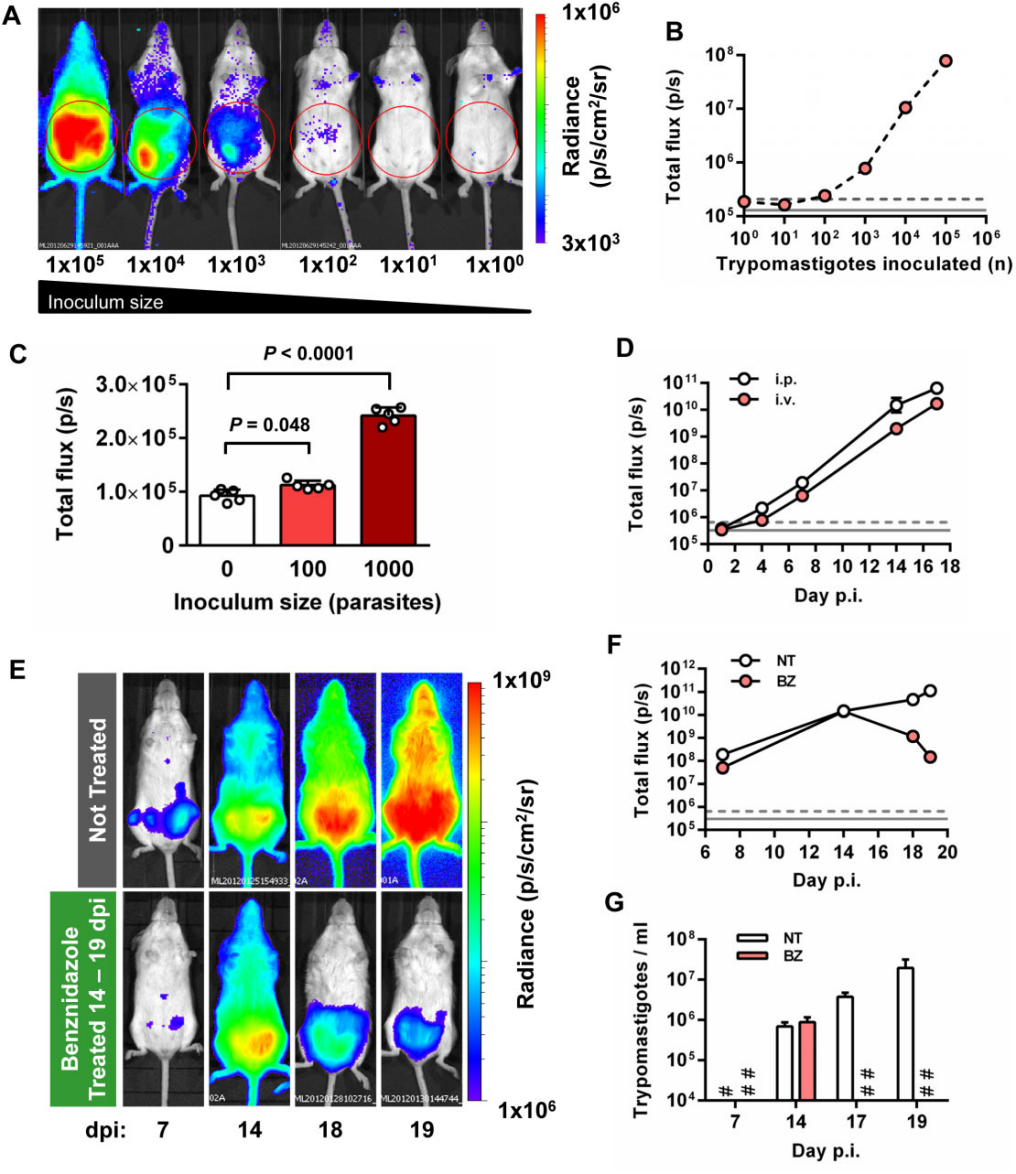
Evaluation of *T. cruzi* infection in SCID mice by *in vivo* bioluminescence imaging.A and B. Evaluation of *in vivo* limit of parasite detection.
Representative ventral images of SCID mice 1 h after i.p. injection with 1 × 10^0^-1 × 10^5^ blood trypomastigotes (BTs) of *PpyRE9h* luciferase-expressing *T. cruzi*. Pseudocolour heat-maps indicate intensity of bioluminescence from low (blue) to high (red). All images use the same log10 scale heat-map, minimum and maximum radiance values as indicated. Red circles indicate regions of interest (ROI) used for signal quantification.Quantification of abdominal bioluminescence for mice in the experiment illustrated in A.Abdominal bioluminescence for mice 1 h after i.p. injection with 0, 100 or 1000 BTs (means ± SD, circles indicate values for individual animals, *n* = 5 per group). *P*-values shown are for one-way anova comparisons between infected groups and the uninfected control group.Whole animal total ventral bioluminescence for SCID mice inoculated via i.p and i.v. routes (data are means ± SD, *n* = 3 per group).Example ventral view images of the same individual SCID mouse 7, 14, 18 and 19 days after i.p. injection with 1 × 10^3^ BTs (upper panels) and comparison with an equivalent infected mouse treated with 100 mg kg^−1^ day^−1^ benznidazole (BZ) for 5 days starting at 14 dpi (lower panels). All images use the same log10 scale heat-map with minimum and maximum radiance values as indicated.Whole animal total ventral bioluminescence for mice in the experiment represented in E (data are means ± SD, *n* = 3 per group, one experiment). BZ, benznidazole treated; NT, not treated.Microscopy-based quantification of peripheral parasitaemia for animals in the experiment represented in E (means ± SD, *n* = 3 per group); limit of detection = 2 × 10^4^ ml^−1^. #, NT below detection limit; ##, BZ below detection limit.B, D and F. Grey lines indicate detection thresholds determined as the mean (solid line) and mean +2SDs (dashed line) of background luminescence of control uninfected mice using abdominal (B) or whole animal (D, F) ROI.

We first assessed infectivity of the bioluminescent *T. cruzi* clone in SCID mice. In this model, both parental WT and bioluminescent parasites caused acute fulminant infections leading to humane end-points being reached 20–24 dpi. The limit of detection *in vivo* was estimated to be close to 100 parasites when mice were imaged 1 h after i.p. injection with blood trypomastigotes (BTs) (Fig. 2A–C), similar to the level of sensitivity achievable in mice infected with *PpyRE9h*-expressing *Trypanosoma brucei* (McLatchie *et al*., 2013). With 1000 parasites and above, there was a linear relationship between inoculum size and whole animal bioluminescence intensity (*R*^2^ = 0.99) (Fig. 2B). This level of sensitivity is substantially higher than seen in previous reports of bioluminescent *T. cruzi* expressing wild-type luciferase, in which detection of parasites was restricted to acute stage infection models (Hyland *et al*., 2008; Canavaci *et al*., 2010; Andriani *et al*., 2011). Quantification of whole animal bioluminescence following either i.p. or i.v. inoculation showed similar inferred parasite loads that increased exponentially over time (Fig. 2D). Treatment of SCID mice with benznidazole (100 mg kg^−1^ day^−1^ for 5 days, starting at 14 dpi) led to a progressive reduction of bioluminescence (Fig. 2E and F), reversion to subpatent parasitaemia (Fig. 2G), and improvements in animal condition, including reduced piloerection and increased mobility. Parasites remained easily detectable by bioluminescence imaging, even when peripheral blood samples were below the detection limit for microscopic examination (Fig. 2E–G). *Post-mortem* histological analysis of tissues from infected SCID mice revealed abundant intracellular amastigote nests in cardiac, skeletal and smooth muscle tissues (Fig. S2). These data clearly demonstrated the utility of *PpyRE9h*-expressing *T. cruzi* as a tool for real-time evaluation of acute infections in immunocompromised mice.

### Chronic *T. cruzi* infections are highly dynamic

**Figure 3:**
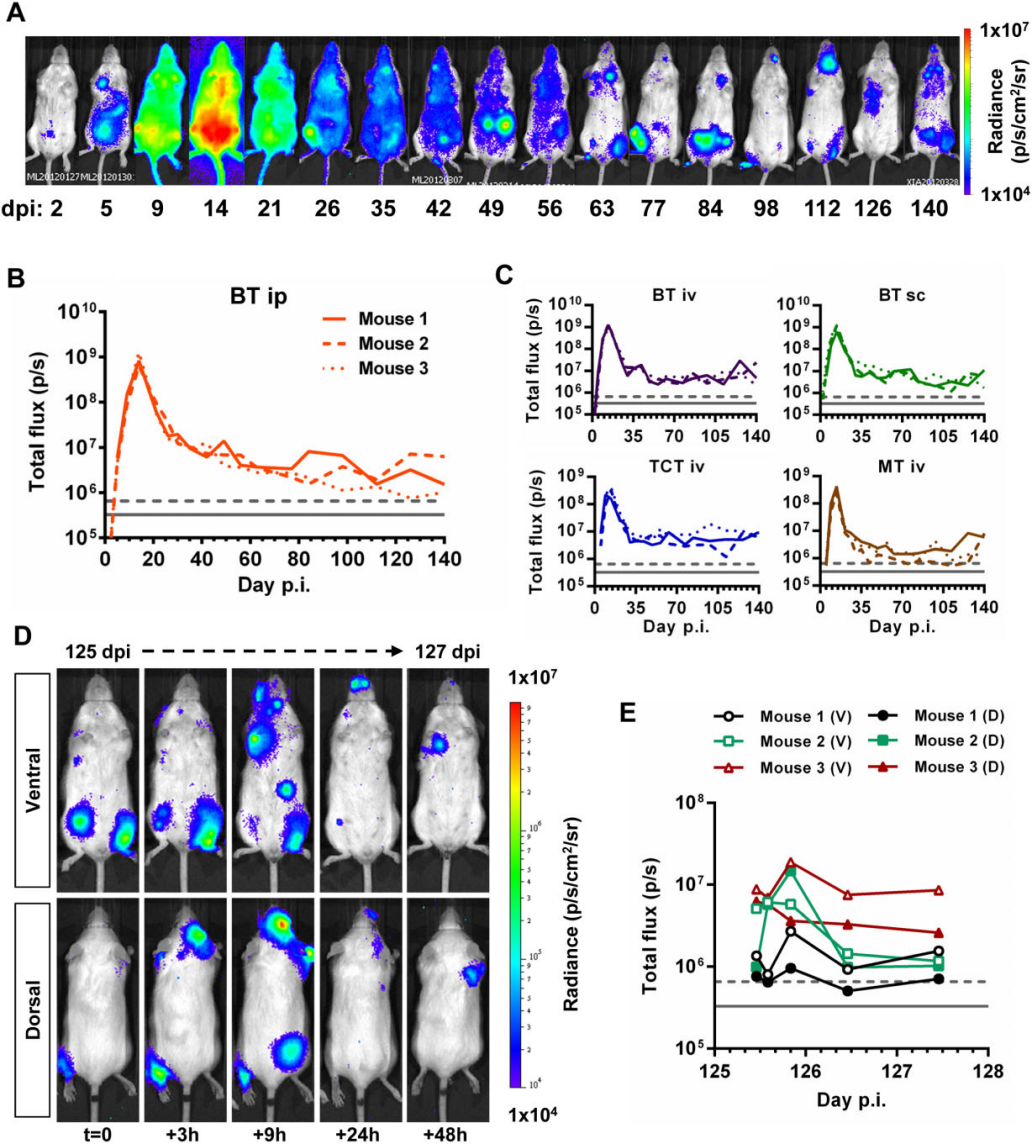
Chronic *T. cruzi* infection is highly dynamic in space and time.
Representative ventral view images of the same BALB/c mouse taken at sequential time points over the course of 140 days after i.p. inoculation with 1 × 10^3^ *PpyRE9h* luciferase-expressing *T. cruzi* CL Brener blood trypomastigotes (BT) (representative of *n* = 3–6 per experiment). Heat-maps are on log10 scales and indicate intensity of bioluminescence from low (blue) to high (red); the minimum and maximum radiances for the pseudocolour scale are indicated.Quantification of whole animal total ventral bioluminescence for three individual mice from the experiment represented by the images in A. Grey lines indicate detection thresholds determined as the mean (solid line) and mean +2SD (dashed line) of background luminescence of control uninfected mice.Quantification of whole animal total ventral bioluminescence of mice inoculated with 1 × 10^3^ BTs, tissue culture trypomastigotes (TCT) or metacyclic trypomastigotes (MT) via i.p., i.v. or s.c. routes. Data are shown for three individual mice per group. Grey lines indicate detection threshold limits determined as in B.Paired ventral and dorsal view images of the same individual chronically infected mouse imaged at increased frequency over a 48 h period starting on day 125 pi (representative three mice per experiment). Heat-maps are on log10 scales and indicate intensity of bioluminescence from low (blue) to high (red); the minimum and maximum radiances for the pseudocolour scale are indicated.Quantification of total ventral (V) and dorsal (D) bioluminescence for three individual animals represented by images in D. Grey lines indicate detection threshold limits determined as in B.

As in humans, chronic infections in mice are characterized by microscopically subpatent parasitaemia. Furthermore, techniques that allow parasite burden and distribution to be monitored in real-time over the course of long-term infections have not been reported. The high sensitivity of our bioluminescent reporter parasite system suggested it would allow us to track such infections in individual immunocompetent animals. We found that the bioluminescence profile in i.p. infected BALB/c mice initially followed a pattern similar to that observed in SCID mice, with restriction to the abdomen, followed by rapid dissemination (Fig. 3A). The bioluminescence-inferred parasite burden peaked at 14 dpi and then reduced progressively until stabilizing between 28 and 42 days after infection, reflecting the transition from the acute to the chronic phase. Bioluminescence in individual mice then fluctuated throughout the course of the infection, at a level 100- to 1000-fold lower than the acute phase peak (Fig. 3A–C). Peripheral blood parasitaemia was detectable only at the peak of acute infection (14 dpi), with a maximum of 2.4 × 10^5^ trypomastigotes ml^−1^ (not shown). The courses of infection in mice inoculated with BTs via the i.p., i.v. or s.c. routes did not vary significantly (Fig. 3B and C). Compared with BT inoculations, tissue culture trypomastigotes (TCTs) or metacyclic trypomastigotes (MTs) generated 2.3-fold and 1.5-fold lower acute bioluminescence peaks at 14 dpi respectively but led to similar chronic infection profiles (Fig. 3C).

After resolution of the acute phase, bioluminescence was typically restricted to between one and five discrete foci per animal, the spatial distribution of which was highly variable (Fig. 3A and D and Fig. S3). Sites frequently associated with bioluminescent foci, included the abdomen, mouth/snout, and positions consistent with a variety of lymph nodes, e.g. cervical, inguinal, axial and mastoid sites. Bioluminescence associated with the position of the heart was rarely observed. In parallel to the frequent fluctuations in whole body bioluminescence, the magnitude of signal from individual foci was also highly dynamic between time points, with foci appearing and disappearing over the course of a single day; few foci were detectable for more than 24 h (Fig. 3D and E and Fig. S3). The same spatially and quantitatively dynamic distribution of bioluminescence during chronic infection was observed irrespective of inoculation route (i.p., i.v. or s.c.) or infective form (BT, TCT or MT). Since the CL Brener strain amastigote doubling time is 16–20 h and the intracellular cycle is 6–8 days, this spatio-temporal pattern is unlikely to reflect parasites replicating *in situ* within distinct tissues sites. Rather, the data suggest that a large proportion of the chronic parasite burden detectable by whole animal bioluminescence imaging is likely to be due to infected host cells being trafficked around the body. Given the capacity of this system to detect as few as 100 parasites dispersed within the peritoneal space (Fig. 2C), the distinct bioluminescent foci observed in the chronic phase (Fig. 3A and D and Fig. S3) may well correspond to single infected cells, which can contain up to 500 amastigotes and would act as a point source of bioluminescence.

### The gut is the primary site of parasite persistence in the BALB/c model of chronic Chagas disease

To investigate *T. cruzi* tissue tropism in the absence of adaptive immunity, *ex vivo* bioluminescence imaging was conducted on different organs and tissue samples from SCID mice with fulminant acute infections. All tissue types obtained from these mice were found to display detectable bioluminescence (Fig. S2). Quantification of tissue/organ-specific radiance, which is a measure of bioluminescence per unit of surface area, showed that in this immunodeficient host, the highest inferred relative parasite loads were present in visceral fat deposits, the uterus, ovaries, gut mesenteries, and the spleen (Fig. S2). The high relative abundance of parasites in these tissues compared to other sites occurred in both i.p and i.v. inoculated SCID mice (Fig. S2).

**Figure 4:**
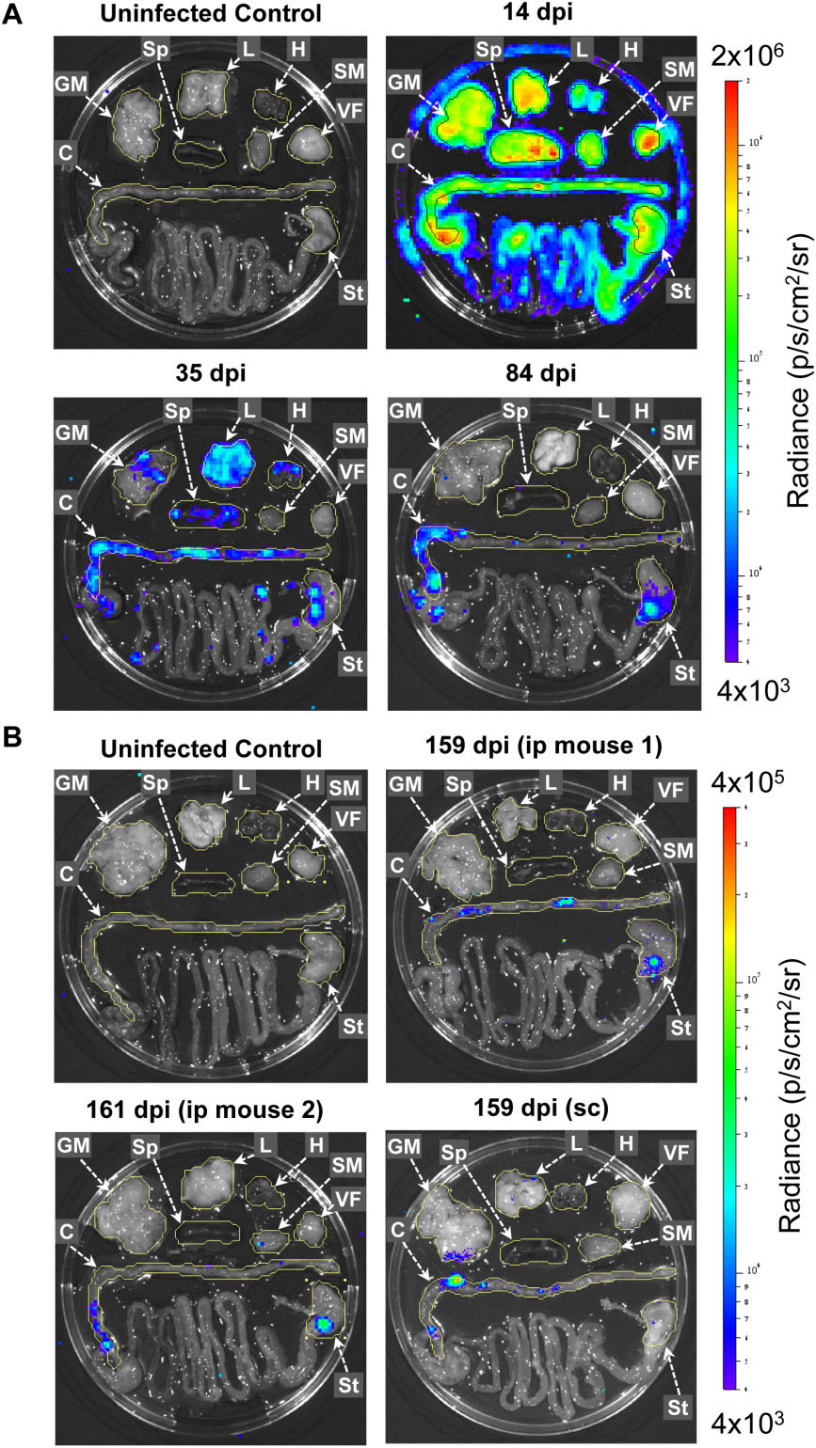
Tissue-specific *ex vivo* imaging identifies the gut as the primary site of *T. cruzi* persistence.A and B. Representative *ex vivo* bioluminescence images for selected organs and tissues taken from BALB/c mice at different times after infection with 1 × 10^3^ *PpyRE9h* luciferase-expressing *T. cruzi* CL Brener parasites. Animals were perfused with 0.3 mg ml^−1^ d-luciferin in PBS via the heart immediately *post-mortem*, then samples were excised and incubated in 0.3 mg ml^−1^ d-luciferin in PBS during bioluminescence evaluation in an *in vivo* imaging platform. Heat-maps are on log10 scales and indicate intensity of bioluminescence from low (blue) to high (red); the minimum and maximum radiances for the pseudocolour scale are indicated. Yellow (uninfected control and 35, 84, 159 and 161 dpi) or black (14 dpi) lines demarcate regions of interest used to quantify tissue-specific bioluminescence. C, colon; H, heart; L, lung; GM, gut mesenteries; SM, skeletal muscle; Sp, spleen; St, stomach; VF, visceral fat.
Representative images for *ex vivo* analysis at 14, 35 and 84 days after i.p. inoculation and for uninfected control. Note the high bioluminescence signals at 14 dpi generate detectable reflection at the Petri dish edge but not at later time points.Representative images for *ex vivo* analysis at 159–161 days after i.p. inoculation,159 days after s.c. inoculation and for uninfected control.

**Figure 5:**
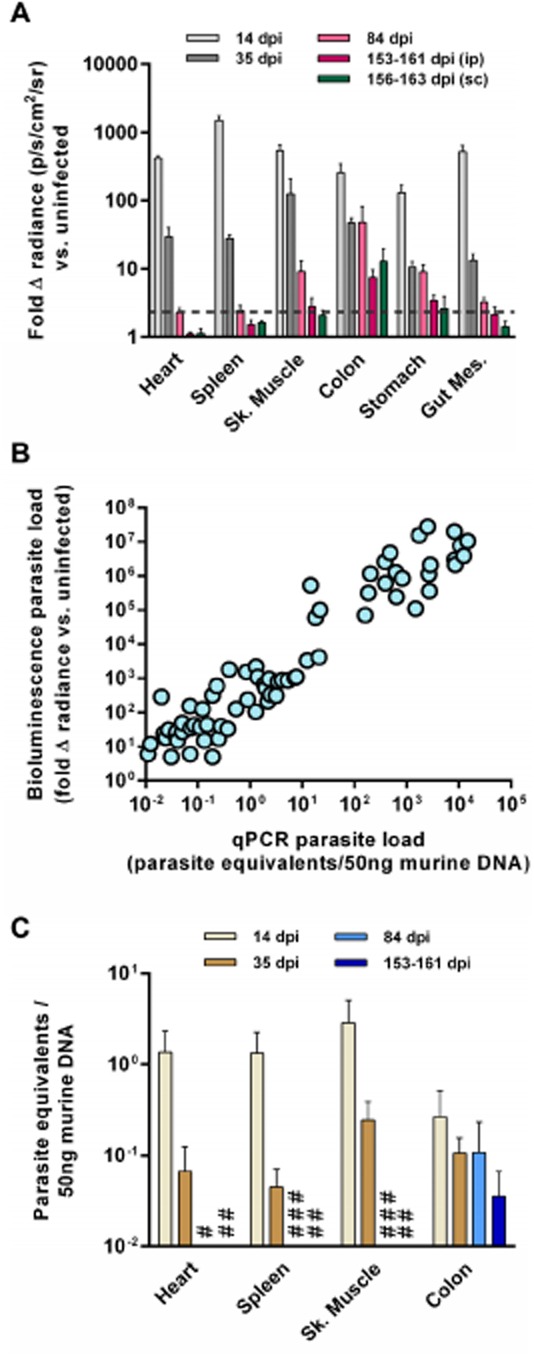
Quantification of tissue-specific parasite loads in *T. cruzi*-infected BALB/c mice.A and B. Quantification of *ex vivo* bioluminescence for selected organs and tissues taken immediately *post-mortem* from BALB/c mice infected with *PpyRE9h* luciferase-expressing *T. cruzi*.
Animals were inoculated by i.p. injection of 1 × 10^3^ trypomastigotes and *ex vivo* imaging was performed at 14 dpi (*n* = 8), 35 dpi (*n* = 9), 84 dpi (*n* = 9) and 153–161 dpi (*n* = 8). *Ex vivo* imaging was also performed at 156–163 dpi on mice that had been inoculated by s.c. injection of 1 × 10^3^ trypomastigotes (*n* = 6). Data are means + SEM of the fold-change in bioluminescence intensity for organs from infected mice compared with matching organs from uninfected mice and are pooled from two independent experiments (*n* ≥ 3 mice per time point per experiment). Dashed line indicates the detection threshold equal to the mean +2SDs of the fold-change in bioluminescence intensity for empty regions of interest (ROI) in the images obtained for 153–160 dpi (ip) infected mice compared with empty ROI in the images obtained for uninfected mice.Correlation between *ex vivo* bioluminescence-inferred and qPCR-inferred parasite loads in individual tissues samples (*n* = 70 samples from 20 mice from four independent experiments).qPCR analysis of tissue-specific parasite burdens in mice inoculated i.p and sacrificed 14, 35, 84 and 153–161 dpi (*n* = 3–4 per group, one experiment). DNA was extracted from tissue samples and the relative amounts of *T. cruzi* DNA and murine DNA were quantified by real-time PCR amplification of a parasite locus (195 bp satellite) and a mouse control gene (*gapdh*). #, 84 dpi below detection limit; ##, 153–161 dpi below detection limit; ###, 84 dpi not done.

To identify sites of *T. cruzi* persistence in a chronically infected immunocompetent host, *ex vivo* imaging was conducted at different stages of infection in BALB/c mice. This revealed large spatiotemporal changes in tissue-specific parasite burdens over time, not all of which had been evident from whole animal imaging (Figs 3A and D and 4A and B, Figs S3 and S4). At the peak of acute infection (14 dpi), parasite bioluminescence was detectable in all tissues examined, with the highest parasite loads associated with visceral and subcutaneous adipose deposits (Figs 4A and 5A and Fig. S4). Around the acute-chronic transition (35 dpi), the average parasite burden had decreased dramatically in most tissues (Figs 4A and 5A and Fig. S4). In pilot experiments, *ex vivo* imaging analyses of chronically infected mice showed that bioluminescence was not detectable in most organs. Therefore, to minimize necropsy time and so maximize parasite detection sensitivity, we chose to focus on a subset of tissues, including those most relevant to human Chagas disease, namely the heart and GIT, as well as other candidate reservoir sites including skeletal muscle and adipose tissue. At chronic infection time points (84 and 153–161 dpi), the parasite burden was almost completely restricted to the GIT, specifically the colon and the stomach, with minor bioluminescence signals associated with the skeletal muscle or gut mesenteries in some animals (Figs 4B and 5A). We considered that our use of i.p. injection to inoculate parasites might represent a confounding factor affecting tissue distribution of *T. cruzi*, even after several months. However, *ex vivo* analysis of tissues from mice 156–163 days after subcutaneous inoculation also demonstrated that bioluminescence was consistently detected in the GIT and in no other tissue (Figs 4B and 5A).

To validate bioluminescence as an accurate proxy for parasite burden, we used tissue-specific qPCR to quantify *T. cruzi* DNA (190 bp satellite locus) in tissue samples from infected mice that had been analysed by *ex vivo* imaging. Targeting this locus, present at ∼ 10 000 copies per cell, can permit detection of a single parasite in 1 ml of blood (Piron *et al*., 2007). There was a clear correlation between qPCR-inferred and bioluminescence-inferred parasite loads (Fig. 5B). Importantly, comparison of tissue-specific qPCR-inferred parasite loads confirmed the observations made with bioluminescence, including the key findings that in chronic infections, the GIT was the main site of persistence and parasites could not be detected in the heart (Fig. 5C). Indeed, extrapolating from the qPCR data at 84 and 153–161 dpi, we estimated that there was less than 1 parasite equivalent per 10^6^ heart cells, indicating that this organ is essentially parasite-free in this infection model. Taken together these data clearly show that the GIT, the colon and stomach in particular, represents the primary site for long-term parasite persistence in immunocompetent BALB/c mice infected with the CL Brener *T. cruzi* strain.

### Development of chronic phase cardiac pathology in the absence of detectable parasite load

**Figure 6:**
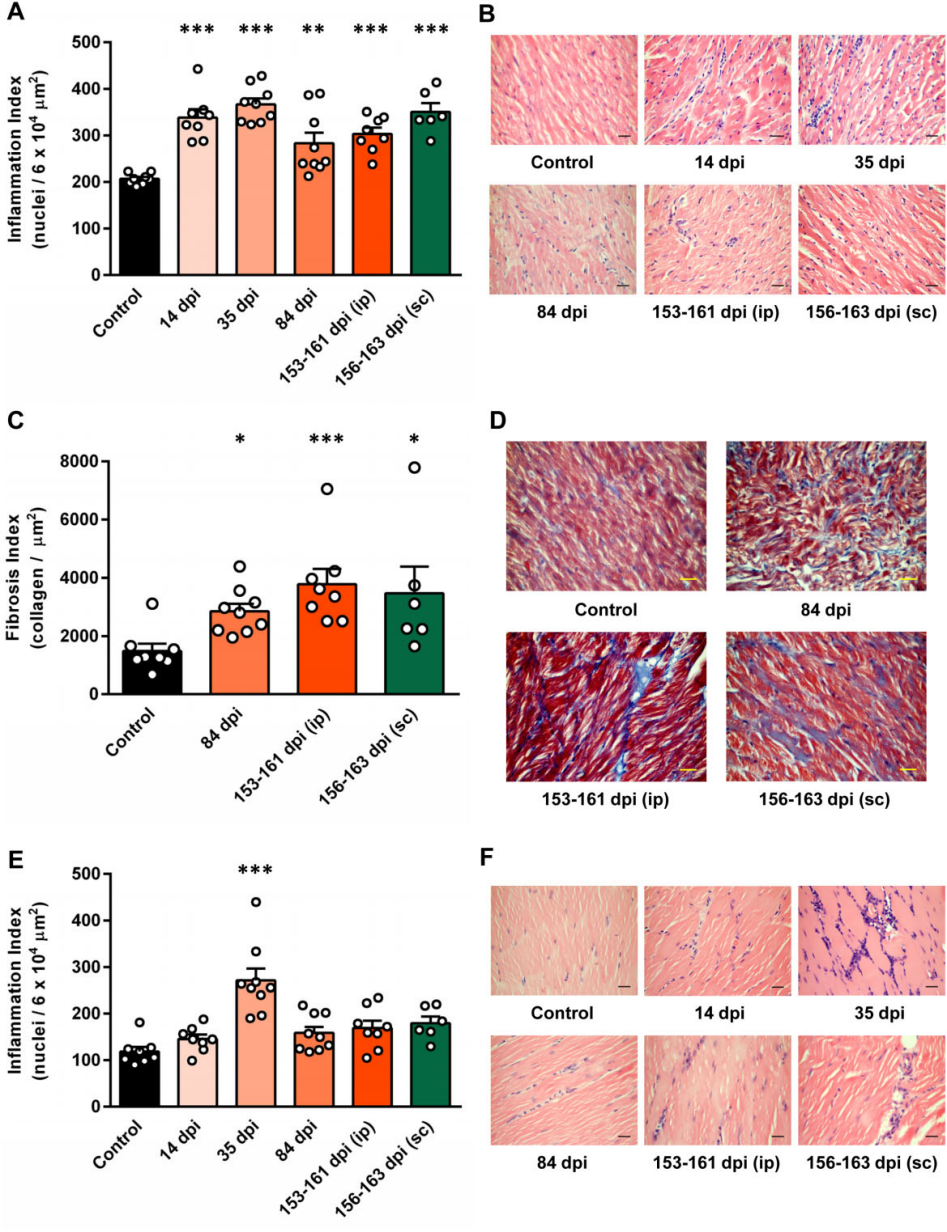
Histological evidence for chronic cardiac inflammation and fibrosis in *T. cruzi* infected BALB/c mice. Cardiac and skeletal muscle samples were obtained at acute and chronic stages of infection after inoculation with 1 × 10^3^ trypomastigotes via i.p. [14 (*n* = 8), 35 (*n* = 9), 84 (*n* = 9) and 153–161 (*n* = 8) dpi] or s.c. [156–163 dpi only (*n* = 6)] injection and from uninfected control mice (*n* = 8) from the same cohorts as the 153–163 dpi animals, then processed for histopathological analysis.
Quantitative analysis of myocarditis. The level of cellular infiltration into cardiac muscle was assessed by automated counting of the number of nuclei in haematoxylin-eosin stained cardiac muscle sections from *T. cruzi* infected and uninfected control mice to derive an inflammation index (nuclei per 6 × 10^4^ μm^2^).Representative images of cardiac muscle from mice at different stages of infection compared with uninfected controls (haematoxylin-eosin stain).Quantitative analysis of cardiac fibrosis. The level of fibrosis in cardiac muscle was assessed by automated counting of the area of collagen (blue) in cardiac muscle sections stained with Masson's trichrome from *T. cruzi* infected and uninfected control mice to derive a fibrosis index (collagen per μm^2^).Representative images of cardiac muscle from mice at different stages of infection compared with uninfected controls (Masson's trichrome stain).Quantitative analysis of skeletal muscle inflammation. The level of cellular infiltration into skeletal muscle was assessed as for cardiac muscle.Representative images of skeletal muscle from mice at different stages of infection compared with uninfected controls (haematoxylin-eosin stain). A, C and E. Data are the means + SEM of six to nine mice per time point pooled from two independent experiments (*n* ≥ 3 mice per time point per experiment); circles indicate values for individual animals. Asterisks indicate *P*-values for one-way anova (A, E) or Kruskal–Wallis (C) comparisons between infected groups and uninfected control groups (&*P* < 0.05; &&*P* < 0.01; &&&*P* < 0.001).

We next aimed to address the question of whether chronically infected mice displayed chagasic heart disease pathology in the absence of detectable, locally persistent *T. cruzi* infection. Cardiac muscle in infected BALB/c mice consistently displayed significant levels of diffuse inflammatory mononuclear cell infiltration (Fig. 6A and B), as well as mild oedema throughout the course of infection. Significant deposition of collagen in heart tissue sections indicated the development of fibrosis in chronic infections (Fig. 6C and D). BALB/c skeletal muscle sections displayed evidence of inflammation and interstitial oedema at all the time points, although this was only significant at 35 dpi (*P* < 0.0001) (Fig. 6E and F). However, in contrast to cardiac muscle, skeletal muscle showed no evidence of fibrosis (not shown). Parasites were not observed in histological sections of heart, skeletal muscle or GIT samples from infected BALB/c mice at any time point. Thus, *T. cruzi* infected BALB/c mice displayed chronic myocarditis and cardiac fibrosis, which are hallmarks of chagasic heart disease, despite the absence of parasites in the heart as measured by histology, bioluminescence imaging or highly sensitive tissue-specific qPCR.

## Discussion

One of the major goals of Chagas disease research has been to gain a better understanding of infection dynamics during chronic stage infections, and their association with the development of pathological outcomes. Progress in this area has been limited because of the scarcity of *T. cruzi* in blood and the difficulty of sampling tissue parasites with existing detection methods. Employing a new, highly sensitive bioluminescence imaging model, we were able to track the course of *T. cruzi* infection in individual BALB/c mice for many months and quantify tissue-specific parasite burdens at different stages of infection through *post-mortem ex vivo* imaging. The CL Brener clone used in this study has been considered ‘myotropic’, because parasites have consistently been observed in heart and muscle tissues during the acute phase of infection (Melo and Brener, 1978; Waghabi *et al*., 2002; Roffê *et al*., 2006; Rodrigues *et al*., 2010). However, comprehensive histological analysis has demonstrated ubiquitous tissue distribution in CL Brener acute infections, with highest parasite loads in adipose tissue (Lenzi *et al*., 1996). Our data corroborated this widespread tissue distribution of parasites during the acute phase in immunocompromised and immunocompetent models and indicated that tissues with high adipose content harboured the highest parasite loads (Figs 4A and 5A, and Fig. S4). The tissue distribution of *T. cruzi* during the chronic stage was strikingly different. We found that resolution of acute infections involves efficient parasite clearance from most tissue types, including the heart and adipose, resulting in long-term persistent infections characterized by a highly restricted distribution of parasites. *Ex vivo* imaging showed that the GIT is the only site that consistently acts as a parasite reservoir in this murine model. In particular, the colon and/or the stomach were always associated with bioluminescent foci. Chronic phase *ex vivo* GIT bioluminescence was typically highly focal, but displayed relatively low variation in intensity between mice, indicating a numerically stable but spatially dynamic parasite load in this organ.

Data on the presence of *T. cruzi* in the gut in humans is limited. Histological investigations have identified persistence of parasites in GIT samples in 20–50% of mega-oesophagus cases (Adad *et al*., 1991; de Castro Côbo *et al*., 2012), but using PCR-based strategies other authors have found *T. cruzi* DNA in 100% of such samples (da Silveira *et al*., 2005). Nevertheless, anti-parasitic chemotherapy has not been previously considered justifiable for seropositive individuals with digestive symptoms, but normal heart function (Bern, 2011). This is primarily due to a lack of evidence for the efficacy of treatment in improving the outcome of digestive forms of chronic Chagas disease, but is also influenced by the prevailing view that megasyndromes are the result of denervation of the GIT during the acute phase of infection (Köberle, 1970; de Oliveira *et al*., 1998). Under this hypothesis, initial denervation in the gut is caused by iNOS-mediated collateral destruction during acute inflammatory responses to local infection (Arantes *et al*., 2004). Further age-related denervation is thought to gradually unmask the parasite-driven losses, leading to progressive organ dilatation and dysfunction. Our finding of long-term persistence of *T. cruzi* in the GIT raises the possibility that local infection may continue to influence the development of digestive forms of Chagas disease into the chronic phase. An intriguing question is the extent to which gut-resident parasites may also influence disease pathogenesis at distant sites, such as the heart. The bioluminescence imaging model used in this study will be a valuable tool for investigating this and other questions relating to the significance of *T. cruzi* sequestration in the gut.

A second feature of *T. cruzi* infection is our observation of large quantitative variations in whole animal bioluminescence, coupled with highly dynamic spatial variation of bioluminescence foci, both occurring at a frequency of less than 24 h (Fig. 3 and Fig. S3). Interpretation of this finding is complicated by the fact that the detection threshold is dependent on the three-dimensional position of parasitized cells. The same number of parasites will generate a stronger signal from peripheral sites than from within deep tissue. This could explain why abdominal foci were transient by *in vivo* imaging (Fig. 3 and Fig. S3), while the *ex vivo* data always demonstrated chronic infection in the gut (Figs 4 and 5). Anatomical movement of the GIT might explain some slight spatial fluctuations in the abdominal region, but we also observed dynamic foci in the thoracic area and regions of the head. In some cases, the rapid appearance and disappearance of bioluminescent foci may derive from visualization of mature pseudocysts prior to rupture in sites where only the final stages of the intracellular cycle of amastigote multiplication are above the detection threshold. The most parsimonious interpretation, however, is that during chronic infection, mobile host cells harbouring parasites, presumably phagocytes, continually traffic to and from peripheral sites. Such movement of infected cells between sites that have different detection thresholds, combined with variation in the parasite load of individual cells would explain the spatiotemporal dynamism in the data. This hypothesis is further supported by the frequent association of bioluminescence foci with the anatomical positions of lymph nodes and our subsequent ability, when informed by bioluminescence, to derive parasite cultures from these nodes. The data presented indicate that parasites do traffic to sites outside the GIT but are unable to maintain long-term foci of infection outside this site. Such continual but transient migration to peripheral sites may represent an adaptation that facilitates uptake by haematophagous triatomine vectors. This process may also have a role in the association between immunosuppression and the development of rare forms of Chagas disease such as meningoencephalitis (Ferreira *et al*., 1997) and cutaneous plaques (Riganti *et al*., 2012).

Restriction of *T. cruzi* to the GIT and transient escape to other sites implies a model of long-term parasite persistence where the mammalian host is able to mount effective systemic anti-parasite responses, but that *T. cruzi* is able to evade these within the gut niche. This is analagous to the strategy employed by *Leishmania major*, which establishes chronic asymptomatic infection in the skin after resolution of acute cutaneous lesions, a process mediated by IL-10 and regulatory T cells (Belkaid *et al*., 2001; 2002[Bibr b8],[Bibr b9]; Anderson *et al*., 2007). Like the skin, the immunological microenvironment of the mammalian gut is generally anti-inflammatory and tolerogenic due to the constitutive presence of commensal microbes and food antigens against which inflammatory responses would be counter-productive (Macpherson and Smith, 2006; Murai *et al*., 2009). Features of the gut microenvironment, including high levels of IL-10, abundant regulatory T cells and macrophage populations that are refractory to activation (Smythies *et al*., 2005) may therefore create a permissive niche for long-term *T. cruzi* persistence. It is also likely that the parasite can actively modulate host cell phenotypes to its long-term benefit, for example, via multiple enzymatic antioxidant strategies (Piacenza *et al*., 2009) or inhibition of antigen presentation (La Flamme *et al*., 1997).

Variation in Chagas disease presentation, progression and differential organ involvement is assumed to be influenced by both host and parasite factors (Dutra and Gollob, 2008), yet very few candidate mechanisms have been identified. There is a consensus that development and progression of CCC is dependent on the continuous presence of *T. cruzi* in the infected host and that chronic inflammation in the heart is the primary feature that drives tissue damage (Marin-Neto *et al*., 2007; Gutierrez *et al*., 2009). The proposed association between persistence of parasites in the heart and development of cardiomyopathy is based on the detection of *T. cruzi* antigens and genomic material in chagasic hearts from humans (Higuchi *et al*., 1993; Jones *et al*., 1993; Añez *et al*., 1999) and experimentally infected animals (Zhang and Tarleton, 1999). However, a quantitative correlation between ongoing cardiac parasite burden and disease progression has yet to be established. An intensive histopathological analysis of human chagasic hearts failed to find a correlation between antigen levels and markers of disease severity (Palomino *et al*., 2000). The severity of CCC in C57BL/6 mice infected with the *T. cruzi* Colombian strain, as determined by serum creatine kinase cardiac isoenzyme MB activity and electocardiograph abnormalities, was also found not to have any association with local parasite burden or myocarditis intensity (Silverio *et al*., 2012). The fact that anti-trypanosomal chemotherapy may improve disease outcome does support the requirement of parasite persistence for pathology, but it cannot give insight into organ-specific effects. Lastly, nucleic acid and antigen detection methods do not demonstrate the presence of live parasites – a limitation that can be addressed by the use of bioluminescent reporter parasites, as in this study. Our data challenge the idea that chronic parasitism of the myocardium itself directly sustains pathogenic myocarditis. Heart-specific bioluminescence was not detected in chronic stage infections, yet we observed myocarditis and diffuse fibrosis, which are hallmarks of CCC in humans. Although we cannot completely exclude the possibility of transient cardiac parasitism, this was not detectable by *in vivo* or *ex vivo* imaging, tissue-specific qPCR, or histological analysis. The finding of an apparent inverse correlation between parasite burden and accumulation of fibrosis in the heart could be due to tissue damage linked to immune-mediated clearance of local parasites. However, several aspects of the data are inconsistent with such an interpretation. Inflammation was observed in skeletal muscle but fibrosis did not develop, despite longer-lasting persistence of parasites in this tissue. The intensity of cardiac inflammation was elevated throughout the infection and so did not correlate with heart-specific parasite load and, crucially, continued in hearts that were free of detectable parasites. Moreover, the relative reduction in heart-specific parasite load between 84 dpi and 153 dpi was negligible compared to the decrease between 14 dpi and 84 dpi, yet these periods were associated with approximately equal levels of collagen deposition. Our data are therefore more consistent with a model of CCC development in which the pathologically relevant inflammation of the cardiac muscle is not dependent on parasites residing continuously in the myocardium. This does not imply that autoimmunity is the sole explanation for ongoing damage to the heart. As mentioned above, we cannot exclude transient, but important episodes of direct cardiac tissue infection or infiltration into the heart by antigen presenting cells infected with *T. cruzi*. Indeed, a recent study has shown that heart-specific parasite burdens in a fatal acute infection model were critically dependent on the ability of *T. cruzi* to replicate in myeloid cells, and not on systemic parasitaemia (Calderón *et al*., 2012), suggesting these cells were trafficking parasites into the heart. This hypothesis fits with our observation of spatiotemporally dynamic foci during the chronic phase of infection. It will therefore be interesting to determine whether such ‘Trojan horse’ models can help to explain parasite dynamics and pathogenesis in chronic infections.

*Trypanosoma cruzi* is a highly diverse species comprised of four major genetic lineages and two inter-lineage hybrid groups (Lewis *et al*., 2011). The severity of heart disease in experimental models is known to vary between different combinations of parasite and mouse strain (Postan *et al*., 1987; Andrade and Magalhães, 1997; Marinho *et al*., 2004; Roffê *et al*., 2012). As demonstrated in this study, bioluminescence imaging technology now has the potential to generate a fuller understanding of long-term infection dynamics for other *T. cruzi* strains and their association with Chagas disease pathogenesis. More broadly, the use of red-shifted luciferase reporters has clear potential to improve *in vivo* imaging models where detection sensitivity is a limiting factor.

## Experimental procedures

### Parasites

The *T. cruzi* strain CL Brener (genotype TcVI), was cultivated as epimastigotes in supplemented RPMI-1640 medium at 28°C as described previously (Kendall *et al*., 1990). Metacyclic trypomastigotes (MTs) were obtained by transferring exponentially growing epimastigotes to transformation medium (Graces-IH) at 4 × 10^6^ parasites ml^−1^ (Isola *et al*., 1986). MTs were harvested after 4–7 days, when typically 70–90% of parasites had differentiated. Tissue culture trypomastigotes (TCTs) were obtained from infected L6 rat skeletal myoblasts grown in supplemented RPMI-1640 medium at 37°C and 5% CO_2_.

### Vector construction and transfection

The rRNA promoter sequence in plasmid pTRIX (Bot *et al*., 2010) was modified to introduce a 5′ AatII site generating pTRIX2. The firefly luciferase gene variant *PpyRE9h* (Branchini *et al*., 2010) was amplified from the plasmid pTb-AMluc-v (McLatchie *et al*., 2013) using primers 5′-GTTTGGATCCATGGAGGACGCCAAGAACATC-3′ and 5′-GTTTCTCGAGTCAGATCTTGCCGCCCTTCT-3′. It was then cloned into pGEM-T Easy vector, excised using BamHI and XhoI (sites underlined) and then inserted into the multiple cloning site of pTRIX2 to produce the plasmid pTRIX2-RE9h. For transfections, 10 μg of pTRIX2-RE9h was digested with AatII and AscI and the 5.85 kb fragment was gel purified (Fig. 1A). A total of 1 × 10^8^ epimastigotes were electroporated with the Amaxa Nucleofector II, using program X-001 and human T-cell nucleofector buffer (Lonza). Transgenic clones with were obtained by selection with 120 μg ml^−1^ G418. Genomic integration was confirmed by Southern blotting.

### *In vitro* assays

The Luciferase Assay System (Promega) was used to assess luciferase expression in G418-resistant clones from *in vitro* cultures. Briefly, 5 × 10^6^ parasites were washed in PBS then lysed in CCLR buffer at 1 × 10^6^ parasites ml^−1^. Ten microlitres of lysate was mixed with 90 μl of substrate and luminescence was measured immediately using a SpectraMax® M3 microplate reader (Molecular Devices). To assess luciferase expression in individual amastigotes, L6 myoblasts grown on glass coverslips were processed for immunofluorescence analysis 5 days after infection. Coverslips were fixed in 2% paraformaldehyde in PBS, permeabilized using 0.1% Triton X-100, then stained with 1:500 goat anti-luciferase pAb (Promega) followed by 1:1000 Alexa488 conjugated donkey anti-goat IgG (Invitrogen). DNA was labelled using 1 μM Hoechst 33342 before mounting with FluorPreserve (Calbiochem). Images were acquired on an LSM 510 confocal microscope (Zeiss).

### Mice and infections

All animal work was carried out under UK Home Office project licence (PPL 70/6997) and was approved by the London School of Hygiene and Tropical Medicine Ethics Committee. All protocols and procedures were conducted in accordance with the UK Animals (Scientific Procedures) Act 1986 (ASPA). BALB/c mice were purchased from Charles River (UK) and CB17 SCID mice were bred in-house. Animals were maintained under specific pathogen-free conditions in individually ventilated cages. They experienced a 12 h light/dark cycle and had access to food and water *ad libitum*. Female mice aged 8–12 weeks were used in all experiments. In standard experiments, 1 × 10^4^ *in vitro* derived TCTs in 0.2 ml PBS were first used to infect SCID mice via i.p. inoculation. Peripheral blood parasitaemias were determined by diluting 3 μl blood in 27 μl red blood cell lysis buffer and counting using a haemocytometer. Blood from SCID mice was then adjusted to 5 × 10^3^ BTs ml^−1^ with PBS. BALB/c mice were infected with 1 × 10^3^ BTs via i.p injection. In some experiments, mice were infected with up to 1 × 10^6^ MT, TCT or BT suspensions in 0.2 ml PBS via i.p., i.v. or s.c. injection. For drug treatments, benznidazole was prepared from powder at 10 mg ml^−1^ in 7% Tween-80, 3% ethanol (vol/vol). Mice were treated with 100 mg kg^−1^ day^−1^ for 5 consecutive days by oral gavage. At experimental end-points mice were sacrificed by ex-sanguination under terminal anaesthesia.

### Bioluminescence imaging

Mice were injected with 150 mg kg^−1^ d-luciferin i.p., then anaesthetized using 2.5% (vol/vol) gaseous isofluorane in oxygen. To measure bioluminescence, mice were placed in an IVIS Lumina II system (Caliper Life Science) and images were acquired 10–20 min after d-luciferin administration using LivingImage 4.3. Exposure times varied between 1 s and 5 min, depending on signal intensity. After imaging, mice were revived and returned to cages. For *ex vivo* imaging, mice were injected with 150 mg kg^−1^ d-luciferin i.p., then sacrificed by ex-sanguination under terminal anaesthesia 7 min later. Mice were perfused with 10 ml 0.3 mg ml^−1^ d-luciferin in PBS via the heart. Organs and tissues were excised, transferred to a Petri dish or culture dish, soaked in 0.3 mg ml^−1^ d-luciferin in PBS, and then imaged as per live mice. To estimate parasite burden in live mice, regions of interest (ROIs) were drawn using LivingImage v.4.3 to quantify bioluminescence expressed as total flux (photons/second). The detection threshold for *in vivo* imaging was estimated using whole animal ROI data (*n* = 25) for control uninfected mice obtained on 11 different days. To estimate parasite load in *ex vivo* tissues, individual ROIs were drawn to quantify bioluminescence expressed as radiance (photons second^−1^ cm^−2^ sr^−1^). Because different tissue types from uninfected control mice were found to have slightly different background radiances, we normalized the data from infected mice using matching tissues from uninfected controls and used the fold-change in radiance compared to these tissue-specific controls as the final measure of *ex vivo* bioluminescence. The detection threshold for *ex vivo* imaging was estimated using the fold-change in radiance for empty ROIs in images obtained for infected mice compared with matching empty ROIs in images for uninfected control mice.

### Histology

Tissue samples were fixed in 10% buffered formalin for 48–72 h, dehydrated, cleared, and embedded in paraffin. Sections (5 μm) were stained with haematoxylin and eosin or with Masson's trichrome. Histomorphometric studies of inflammation involved analysis of 20 images from randomly selected fields of tissue sections on a single slide per animal. An index of inflammatory infiltration was derived by quantifying the total number of nuclei present in each image (Caliari, 1997). A fibrosis index was derived by quantifying the collagen content of each sample using 30 randomly selected fields for each section stained by Masson's trichrome technique. Blue pixels were counted for the creation of a binary image and posterior calculus of the total area occupied with fibrosis (Caliari *et al*., 2002). All images were taken with a DFC420 light microscope (Leica Microsystems) and analysed using Leica Application Suite software v.4.2 by an investigator blinded to the groups.

### qPCR

Organs and tissues were snap frozen on dry ice and stored at −80°C. For DNA extraction, samples were thawed and immediately homogenized in at least 200 μl lysis buffer (4 M urea, 200 mM Tris, 20 mM NaCl, 200 mM EDTA, pH 7.4) per 50 mg tissue using a BulletBlender Storm instrument (Next Advance). The tissue suspension was then incubated overnight at 37°C with 0.6 mg proteinase K. DNA was extracted using the High Pure PCR product purification kit (Roche). Real-time PCR reactions were prepared using the QuantiTect SYBR Green PCR Kit (Qiagen) and run on a RotorGene 3000 instrument. Each reaction contained 50 ng DNA and 0.5 μM of each primer. *T. cruzi*-specific primers TCZ-F and TCZ-R (Cummings and Tarleton, 2003), targeted at the 195 bp satellite repeat, and mouse-specific primers GAPDHf and GAPDHr (Cencig *et al*., 2011), targeted at the murine *gapdh* gene, were used. Measurements of *T. cruzi* DNA content were normalized using the ratio of Ct values for *T. cruzi*- and mouse-specific PCRs, and converted to estimated numbers of parasite equivalents by reference to a standard curve with a range of 2.5 × 10^6^–2.5 × 10^−1^ parasite equivalents ml^−1^. The standard curve was established from serial dilution of a DNA sample derived from 75 mg homogenized muscle tissue, spiked with 2 × 10^7^ epimastigotes, using DNA from unspiked equivalent samples as the diluent.

### Statistics

Individual animals were used as the unit of analysis of *in vivo* and *ex vivo* experiments. No blinding or randomization protocols were used. Sample sizes were determined empirically. Statistical differences between groups were evaluated using one-way anova with Bonferroni *post-hoc* correction for normally distributed data or using a Kruskal–Wallis test with Dunn's *post-hoc* correction for non-normally distributed data, both in GraphPad Prism v.6. Data sets were tested for departure from normal distribution using the Shapiro-Wilk test in SPSS v.20. Differences of *P* < 0.05 were considered significant.

## Supporting Information

Fig S1 Confirmation of genomic integration of the *PpyRE9h* reporter gene. Schematic illustrating integration of linearized pTRIX2-RE9h into the *T. cruzi* genome by homologous recombination. Integration was confirmed by Southern blot of BamHI (B) and XhoI (X) digested genomic DNA from G418-resistant clones with a radiolabelled *PpyRE9h* ssDNA probe as indicated. The autoradiograph shows data for wild-type (WT) parasites and five transgenic clones (1–5). The probe localized to the expected 8.6 kb BamHI band in all five clones and a variably sized XhoI band indicating integration into different rDNA repeats. Clone 3 was used for all further experiments.

Fig S2 Tissue-specific parasite distributions and loads in acutely infected SCID mice. A. Examples of heart, skeletal muscle and colon samples from SCID mice with fulminant infections showing presence of intracellular amastigotes (arrows). Histological sections were stained with Masson's trichrome (heart) or haematoxylin and eosin (skeletal muscle, colon). Heart and colon: 1000× magnification, scale bar = 30 μm; skeletal muscle: 400× magnification, scale bar = 50 μm. B and C. Quantification of *ex vivo* bioluminescence for selected organs and tissues taken immediately *post-mortem* from SCID mice 17 days after infection with 1 × 10^3^ *PpyRE9h* luciferase-expressing *T. cruzi* trypomastigotes. B. Images of selected organs and tissues from representative uninfected control and infected SCID mice overlaid with bioluminescence heat-map. Both images use the same log10 pseudocolour scale to indicate intensity of bioluminescence from low (blue) to high (red); the minimum and maximum radiances are indicated. Red lines demarcate regions of interest used to quantify tissue-specific bioluminescence. B, brain; C, colon; H, heart; K, kidney; Li, liver; Lu, lung; Me, gut mesenteries; Mu, skeletal muscle; O, ovary; R, ribcage; SF, subcutaneous fat; Sp, spleen; St, stomach; U, uterus; VF, visceral fat. C. Quantification of tissue- and organ-specific bioluminescence for SCID mice inoculated by i.p and i.v. routes (*n* = 3 per group). Data are means + SD of the fold-change in bioluminescence intensity for organs from infected mice compared with matching organs from uninfected mice.

Fig S3 Daily quantitative fluctuation and spatial dynamism of chronic *T. cruzi* infections in BALB/c mice. Ventral and dorsal images of the same three individual BALB/c mice chronically infected with *PpyRE9h* luciferase-expressing *T. cruzi* over the course of 10 days. All images use the same log10 scale heat-map with minimum and maximum radiance values as indicated. Representative of three mice per experiment.

Fig S4 Tissue-specific parasite distributions and loads in acutely infected BALB/c mice. Quantification of *ex vivo* bioluminescence for selected organs and tissues taken immediately *post-mortem* from BALB/c mice infected with *PpyRE9h* luciferase-expressing *T. cruzi*. Animals were inoculated by i.p. injection of 1 × 10^3^ trypomastigotes and *ex vivo* imaging was performed at 14 dpi (*n* = 8, except lung *n* = 7 and peritoneum *n* = 6) and 35 dpi (*n* = 9 for all tissues). Data are means + SD of the fold-change in bioluminescence intensity for organs from infected mice compared with matching organs from uninfected mice and are pooled from two independent experiments (*n* ≥ 3 mice per time point per experiment). Dashed line indicates the detection threshold equal to the mean +2SDs of the fold-change in bioluminescence intensity for empty regions of interest (ROI) in the images obtained for infected mice compared with empty ROI in the images obtained for uninfected mice.

## References

[b1] Adad SJ, Andrade DCDS, Lopes ER, Chapadeiro E (1991). Contribuição ao estudo da anatomia patológica do megaesôfago chagásico. Rev Inst Med Trop Sao Paulo.

[b2] Anderson CF, Oukka M, Kuchroo VJ, Sacks D (2007). CD4+CD25−Foxp3− Th1 cells are the source of IL-10-mediated immune suppression in chronic cutaneous leishmaniasis. J Exp Med.

[b3] Andrade SG, Magalhães JB (1997). Biodemes and zymodemes of *Trypanosoma cruzi* strains: correlations with clinical data and experimental pathology. Rev Soc Bras Med Trop.

[b4] Andriani G, Chessler A-DC, Courtemanche G, Burleigh BA, Rodriguez A (2011). Activity *in vivo* of anti-*Trypanosoma cruzi* compounds selected from a high throughput screening. PLoS Negl Trop Dis.

[b5] Añez N, Carrasco H, Parada H, Crisante G, Rojas A, Fuenmayor C (1999). Myocardial parasite persistence in chronic chagasic patients. Am J Trop Med Hyg.

[b6] Arantes RME, Marche HHF, Bahia MT, Cunha FQ, Rossi MA, Silva JS (2004). Interferon-γ-induced nitric oxide causes intrinsic intestinal denervation in *Trypanosoma cruzi*-infected mice. Am J Pathol.

[b7] Basile L, Jansà JM, Carlier Y, Salamanca DD, Angheben A, Bartoloni A (2011). Chagas disease in European countries: the challenge of a surveillance system. Eurosurveillance.

[b8] Belkaid Y, Hoffmann KF, Mendez S, Kamhawi S, Udey MC, Wynn TA, Sacks DL (2001). The role of interleukin (IL)-10 in the persistence of *Leishmania major* in the skin after healing and the therapeutic potential of anti-IL-10 receptor antibody for sterile cure. J Exp Med.

[b9] Belkaid Y, Piccirillo CA, Mendez S, Shevach EM, Sacks DL (2002). CD4+CD25+ regulatory T cells control *Leishmania major* persistence and immunity. Nature.

[b10] Bern C (2011). Antitrypanosomal therapy for chronic Chagas' disease. N Engl J Med.

[b11] Bern C, Montgomery SP (2009). An estimate of the burden of Chagas disease in the United States. Clin Infect Dis.

[b12] Bonney KM, Engman DM (2008). Chagas heart disease pathogenesis: one mechanism or many?. Curr Mol Med.

[b13] Bot C, Hall BS, Bashir N, Taylor MC, Helsby NA, Wilkinson SR (2010). Trypanocidal activity of aziridinyl nitrobenzamide prodrugs. Antimicrob Agents Chemother.

[b14] Branchini BR, Ablamsky DM, Davis AL, Southworth TL, Butler B, Fan F (2010). Red-emitting luciferases for bioluminescence reporter and imaging applications. Anal Biochem.

[b15] Calderón J, Maganto-Garcia E, Punzón C, Carrión J, Terhorst C, Fresno M (2012). The receptor Slamf1 on the surface of myeloid lineage cells controls susceptibility to infection by *Trypanosoma cruzi*. PLoS Pathog.

[b16] Caliari MV (1997).

[b17] Caliari MV, Machado RDP, de Lana M, Cajá RAF, Carneiro CM, Bahia MT (2002). Quantitative analysis of cardiac lesions in chronic canine chagasic cardiomyopathy. Rev Inst Med Trop Sao Paulo.

[b18] Canavaci AMC, Bustamante JM, Padilla AM, Perez Brandan CM, Simpson LJ, Xu D (2010). *In vitro* and *in vivo* high-throughput assays for the testing of anti-*Trypanosoma cruzi* compounds. PLoS Negl Trop Dis.

[b19] de Castro Côbo E, Silveira TP, Micheletti AM, Crema E, Adad SJ (2012). Research on *Trypanosoma cruzi* and analysis of inflammatory infiltrate in Esophagus and Colon from chronic Chagasic patients with and without mega. J Trop Med.

[b20] Cencig S, Coltel N, Truyens C, Carlier Y (2011). Parasitic loads in tissues of mice infected with *Trypanosoma cruzi* and treated with AmBisome. PLoS Negl Trop Dis.

[b21] Cummings KL, Tarleton RL (2003). Rapid quantitation of *Trypanosoma cruzi* in host tissue by real-time PCR. Mol Biochem Parasitol.

[b22] Cunha-Neto E, Teixeira PC, Nogueira LG, Herbert BT, Kalil J, Louis MW (2011). Autoimmunity. Advances in Parasitology.

[b23] Dutra WO, Gollob KJ (2008). Current concepts in immunoregulation and pathology of human Chagas disease. Curr Opin Infect Dis.

[b24] Ferreira MS, Nishioka SDA, Silvestre MTA, Borges AS, Araújo FRFN, Rocha A (1997). Reactivation of Chagas' disease in patients with AIDS: report of three new cases and review of the literature. Clin Infect Dis.

[b25] Gironès N, Fresno M (2003). Etiology of Chagas disease myocarditis: autoimmunity, parasite persistence, or both?. Trends Parasitol.

[b26] Gutierrez FRS, Guedes PMM, Gazzinelli RT, Silva JS (2009). The role of parasite persistence in pathogenesis of Chagas heart disease. Parasite Immunol.

[b27] Higuchi MDL, Gutierrez PS, Aiello VD, Palomino S, Bocchi E, Kalil J (1993). Immunohistochemical characterization of infiltrating cells in human chronic chagasic myocarditis: comparison with myocardial rejection process. Virchows Arch.

[b28] Hyland KV, Asfaw SH, Olson CL, Daniels MD, Engman DM (2008). Bioluminescent imaging of *Trypanosoma cruzi* infection. Int J Parasitol.

[b29] Isola EL, Lammel EM, Gonzalez Cappa SM (1986). *Trypanosoma cruzi*: differentiation after interaction of epimastigotes and *Triatoma infestans* intestinal homogenate. Exp Parasitol.

[b30] Jones EM, Colley DG, Tostes S, Lopes ER, Vnencak-Jones CL, McCurley TL (1993). Amplification of a *Trypanosoma cruzi* DNA sequence from inflammatory lesions in human chagasic cardiomyopathy. Am J Trop Med Hyg.

[b31] Kendall G, Wilderspin AF, Ashall F, Miles MA, Kelly JM (1990). *Trypanosoma cruzi* glycosomal glyceraldehyde-3-phosphate dehydrogenase does not conform to the ‘hotspot’ topogenic signal model. EMBO J.

[b32] Köberle F (1970). The causation and importance of nervous lesions in American trypanosomiasis. Bull World Health Organ.

[b33] La Flamme AC, Kahn SJ, Rudensky AY, Van Voorhis WC (1997). *Trypanosoma cruzi*-infected macrophages are defective in major histocompatibility complex class II antigen presentation. Eur J Immunol.

[b34] Lenzi HL, Oliveira DN, Lima MT, Gattass CR (1996). *Trypanosoma cruzi*: paninfectivity of CL strain during murine acute infection. Exp Parasitol.

[b35] Lewis MD, Llewellyn MS, Yeo M, Acosta N, Gaunt MW, Miles MA (2011). Recent, independent and anthropogenic origins of *Trypanosoma cruzi* hybrids. PLoS Negl Trop Dis.

[b36] McLatchie AP, Burrell-Saward H, Myburgh E, Lewis MD, Ward TH, Mottram JC (2013). Highly sensitive *in vivo* imaging of *Trypanosoma brucei* expressing ‘red-shifted’ luciferase. PLoS Negl Trop Dis.

[b37] Macpherson AJ, Smith K (2006). Mesenteric lymph nodes at the center of immune anatomy. J Exp Med.

[b38] Marin-Neto JA, Cunha-Neto E, Maciel BC, Simoes MV (2007). Pathogenesis of chronic Chagas heart disease. Circulation.

[b39] Marin-Neto JA, Rassi A, Morillo CA, Avezum A, Connolly SJ, Sosa-Estani S (2008). Rationale and design of a randomized placebo-controlled trial assessing the effects of etiologic treatment in Chagas' cardiomyopathy: The BENznidazole Evaluation For Interrupting Trypanosomiasis (BENEFIT). Am Heart J.

[b40] Marinho CRF, Bucci DZ, Dagli MLZ, Bastos KRB, Grisotto MG, Sardinha LR (2004). Pathology affects different organs in two mouse strains chronically infected by a *Trypanosoma cruzi* clone: a model for genetic studies of Chagas' disease. Infect Immun.

[b41] Matos Ferreira AV, Segatto M, Menezes Z, Macedo AM, Gelape C, de Oliveira Andrade L (2011). Evidence for *Trypanosoma cruzi* in adipose tissue in human chronic Chagas disease. Microbes Infect.

[b42] Melo R, Brener Z (1978). Tissue tropism of different *Trypanosoma cruzi* strains. J Parasitol.

[b43] Moncayo Á, Silveira AC (2009). Current epidemiological trends for Chagas disease in Latin America and future challenges in epidemiology, surveillance and health policy. Mem Inst Oswaldo Cruz.

[b44] Murai M, Turovskaya O, Kim G, Madan R, Karp CL, Cheroutre H, Kronenberg M (2009). Interleukin 10 acts on regulatory T cells to maintain expression of the transcription factor Foxp3 and suppressive function in mice with colitis. Nat Immunol.

[b45] Nagajyothi F, Machado FS, Burleigh BA, Jelicks LA, Scherer PE, Mukherjee S (2012). Mechanisms of *Trypanosoma cruzi* persistence in Chagas disease. Cell Microbiol.

[b46] de Oliveira RB, Troncon LE, Dantas RO, Menghelli UG (1998). Gastrointestinal manifestations of Chagas' disease. Am J Gastroenterol.

[b47] Padilla AM, Bustamante JM, Tarleton RL (2009). CD8+ T cells in *Trypanosoma cruzi* infection. Curr Opin Immunol.

[b48] Palomino SAP, Aiello VD, Higuchi ML (2000). Systematic mapping of hearts from chronic chagasic patients: the association between the occurrence of histopathological lesions and *Trypanosoma cruzi* antigens. Ann Trop Med Parasitol.

[b49] Piacenza L, Alvarez MN, Peluffo G, Radi R (2009). Fighting the oxidative assault: the *Trypanosoma cruzi* journey to infection. Curr Opin Microbiol.

[b50] Piron M, Fisa R, Casamitjana N, López-Chejade P, Puig L, Vergés M (2007). Development of a real-time PCR assay for *Trypanosoma cruzi* detection in blood samples. Acta Trop.

[b51] Postan M, Bailey JJ, Dvorak JA, McDaniel JP, Pottala EW (1987). Studies of *Trypanosoma cruzi* clones in inbred mice: III. Histopathological and electrocardiographical responses to chronic infection. Am J Trop Med Hyg.

[b52] Rassi A, de Rezende JM, Luquetti AO, Tibayrenc M, Rassi A, Telleria J (2010). Clinical phases and forms of Chagas disease. American Trypanosomiasis Chagas Disease.

[b53] Riganti J, Maqueda MG, Piñero MCB, Volonteri V, Galimberti RL (2012). Reactivation of Chagas' disease: cutaneous manifestations in two immunosuppressed patients. Int J Dermatol.

[b54] Rodrigues CM, Valadares HMS, Francisco AF, Arantes JM, Campos CF, Teixeira-Carvalho A (2010). Coinfection with different *Trypanosoma cruzi* strains interferes with the host immune response to infection. PLoS Negl Trop Dis.

[b55] Roffê E, Souza ALS, Caetano BC, Machado PP, Barcelos LS, Russo RC (2006). A DNA vaccine encoding CCL4/MIP-1β enhances myocarditis in experimental *Trypanosoma cruzi* infection in rats. Microbes Infect.

[b56] Roffê E, Rothfuchs AG, Santiago HC, Marino APMP, Ribeiro-Gomes FL, Eckhaus M (2012). IL-10 limits parasite burden and protects against fatal myocarditis in a mouse model of *Trypanosoma cruzi* infection. J Immunol.

[b57] da Silveira ABM, Arantes RME, Vago AR, Lemos EM, Adad SJ, Correa-Oliveira R, D'Avila Reis D (2005). Comparative study of the presence of *Trypanosoma cruzi* kDNA, inflammation and denervation in chagasic patients with and without megaesophagus. Parasitology.

[b58] Silverio JC, Pereira IR, Cipitelli MDC, Vinagre NF, Rodrigues MM, Gazzinelli RT, Lannes-Vieira J (2012). CD8+ T-cells expressing interferon gamma or perforin play antagonistic roles in heart injury in experimental *Trypanosoma cruzi*-elicited cardiomyopathy. PLoS Pathog.

[b59] Smythies LE, Sellers M, Clements RH, Mosteller-Barnum M, Meng G, Benjamin WH (2005). Human intestinal macrophages display profound inflammatory anergy despite avid phagocytic and bacteriocidal activity. J Clin Invest.

[b60] Tarleton RL (2003). Chagas disease: a role for autoimmunity?. Trends Parasitol.

[b61] Teixeira ARL, Hecht MM, Guimaro MC, Sousa AO, Nitz N (2011). Pathogenesis of Chagas' disease: parasite persistence and autoimmunity. Clin Microbiol Rev.

[b62] Waghabi MC, Coutinho CMLM, Soeiro MNC, Pereira MCS, Feige J-J, Keramidas M (2002). Increased *Trypanosoma cruzi* invasion and heart fibrosis associated with high transforming growth factor β levels in mice deficient in α2-macroglobulin. Infect Immun.

[b63] Zhang L, Tarleton RL (1999). Parasite persistence correlates with disease severity and localization in chronic Chagas' disease. J Infect Dis.

