# Supplementary figures and images for: Bioluminescence imaging of chronic Trypanosoma cruzi infections reveals tissue-specific parasite dynamics and heart disease in the absence of locally persistent infection
Source: Cell Microbiol. 2014 May 1;16(9):1285–300. doi: 10.1111/cmi.12297 (PMC4190689; doi:10.1111/cmi.12297)

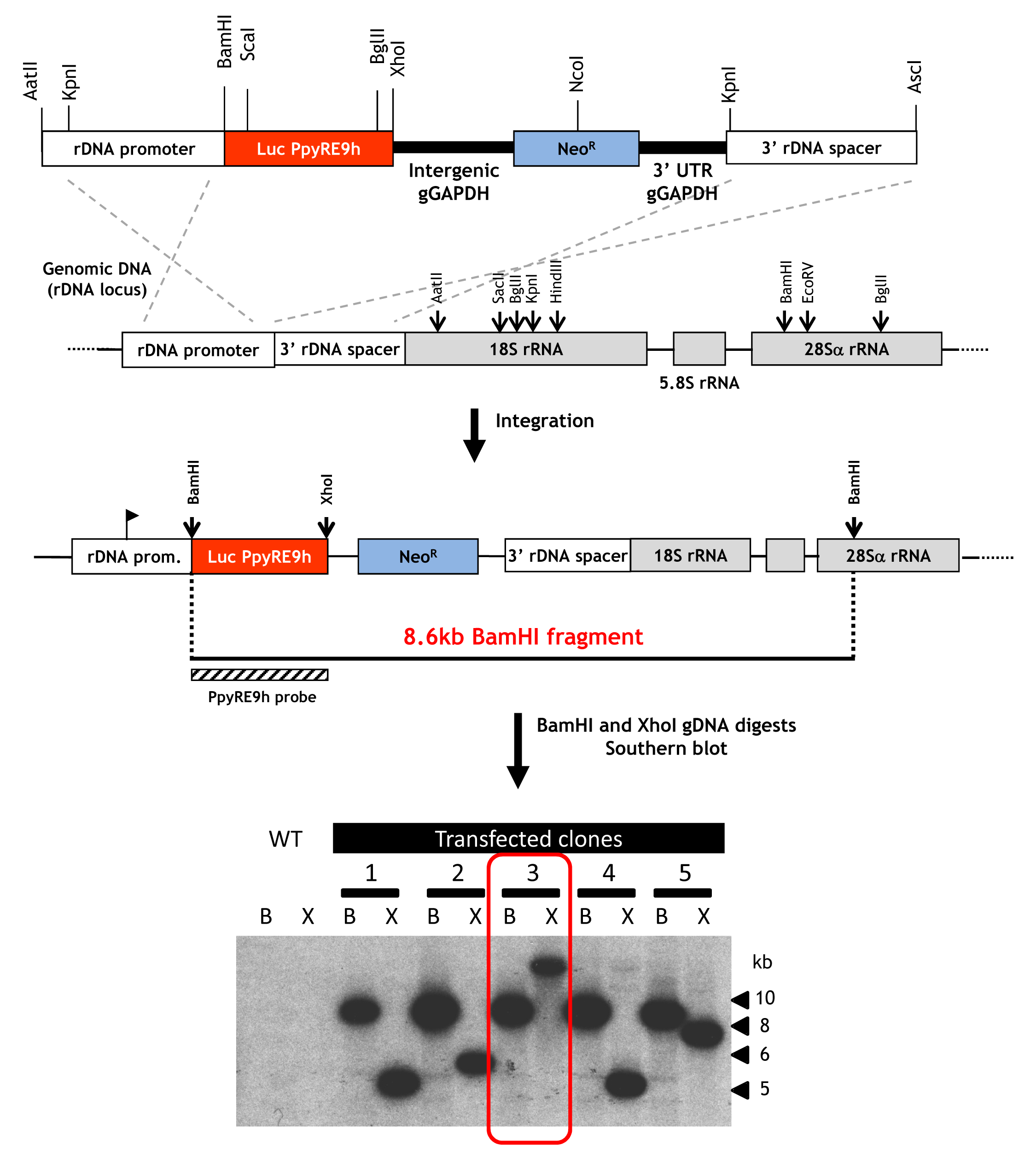

Supplement: Fig S1 — Confirmation of genomic integration of the PpyRE9h reporter gene. Schematic illustrating integration of linearized pTRIX2-RE9h into the T. cruzi genome by homologous recombination. Integration was confirmed by Southern blot of BamHI (B) and XhoI (X) digested genomic DNA from G418-resistant clones with a radiolabelled PpyRE9h ssDNA probe as indicated. The autoradiograph shows data for wild-type (WT) parasites and five transgenic clones (1–5). The probe localized to the expected 8.6 kb BamHI band in all five clones and a variably sized XhoI band indicating integration into different rDNA repeats. Clone 3 was used for all further experiments. [file cmi0016-1285-sd1.tif]

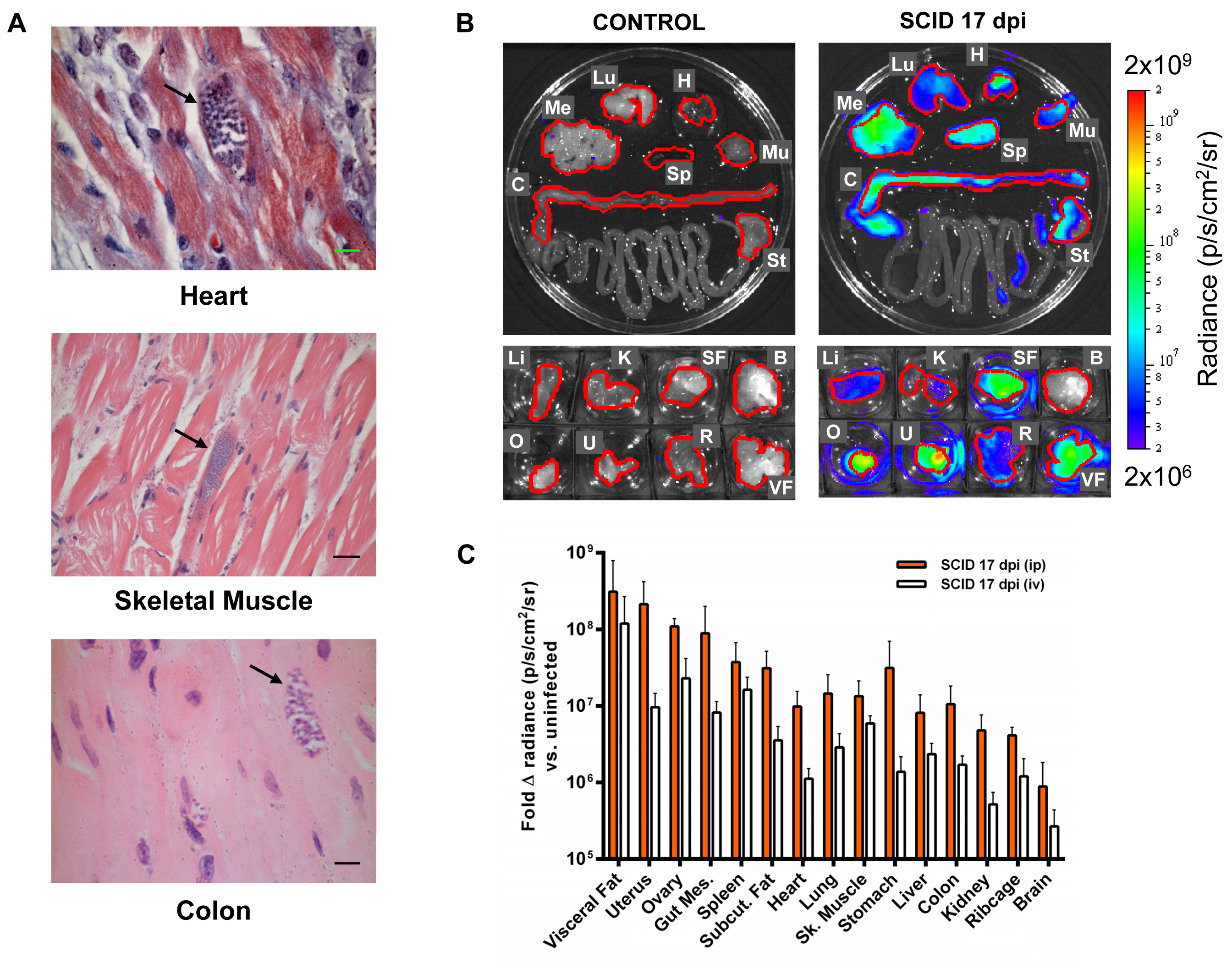

Supplement: Fig S2 — Tissue-specific parasite distributions and loads in acutely infected SCID mice. A. Examples of heart, skeletal muscle and colon samples from SCID mice with fulminant infections showing presence of intracellular amastigotes (arrows). Histological sections were stained with Masson's trichrome (heart) or haematoxylin and eosin (skeletal muscle, colon). Heart and colon: 1000× magnification, scale bar = 30 μm; skeletal muscle: 400× magnification, scale bar = 50 μm. B and C. Quantification of ex vivo bioluminescence for selected organs and tissues taken immediately post-mortem from SCID mice 17 days after infection with 1 × 10^3^ PpyRE9h luciferase-expressing T. cruzi trypomastigotes. B. Images of selected organs and tissues from representative uninfected control and infected SCID mice overlaid with bioluminescence heat-map. Both images use the same log10 pseudocolour scale to indicate intensity of bioluminescence from low (blue) to high (red); the minimum and maximum radiances are indicated. Red lines demarcate regions of interest used to quantify tissue-specific bioluminescence. B, brain; C, colon; H, heart; K, kidney; Li, liver; Lu, lung; Me, gut mesenteries; Mu, skeletal muscle; O, ovary; R, ribcage; SF, subcutaneous fat; Sp, spleen; St, stomach; U, uterus; VF, visceral fat. C. Quantification of tissue- and organ-specific bioluminescence for SCID mice inoculated by i.p and i.v. routes (n = 3 per group). Data are means + SD of the fold-change in bioluminescence intensity for organs from infected mice compared with matching organs from uninfected mice. [file cmi0016-1285-sd2.tif]

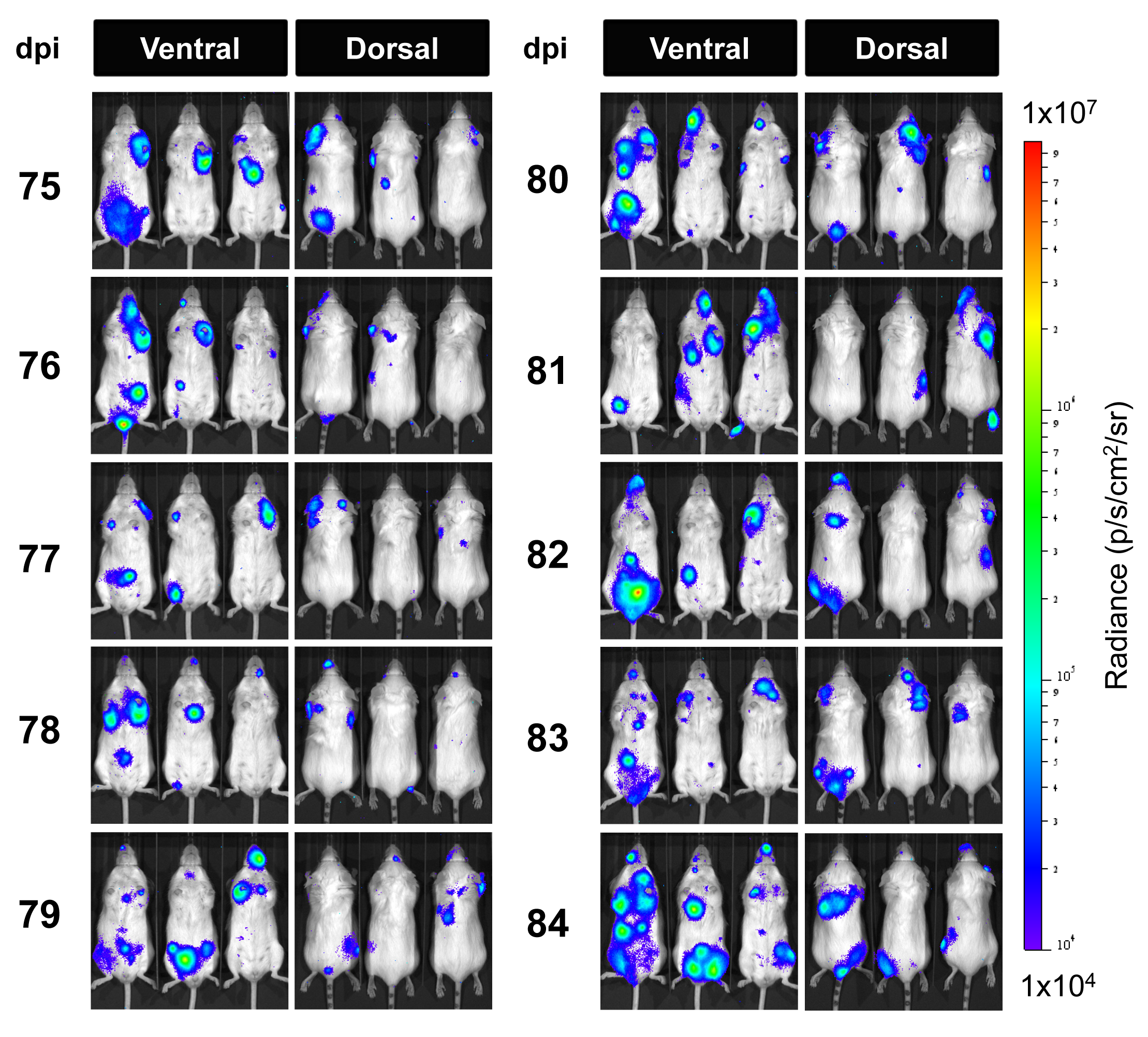

Supplement: Fig S3 — Daily quantitative fluctuation and spatial dynamism of chronic T. cruzi infections in BALB/c mice. Ventral and dorsal images of the same three individual BALB/c mice chronically infected with PpyRE9h luciferase-expressing T. cruzi over the course of 10 days. All images use the same log10 scale heat-map with minimum and maximum radiance values as indicated. Representative of three mice per experiment. [file cmi0016-1285-sd3.tif]

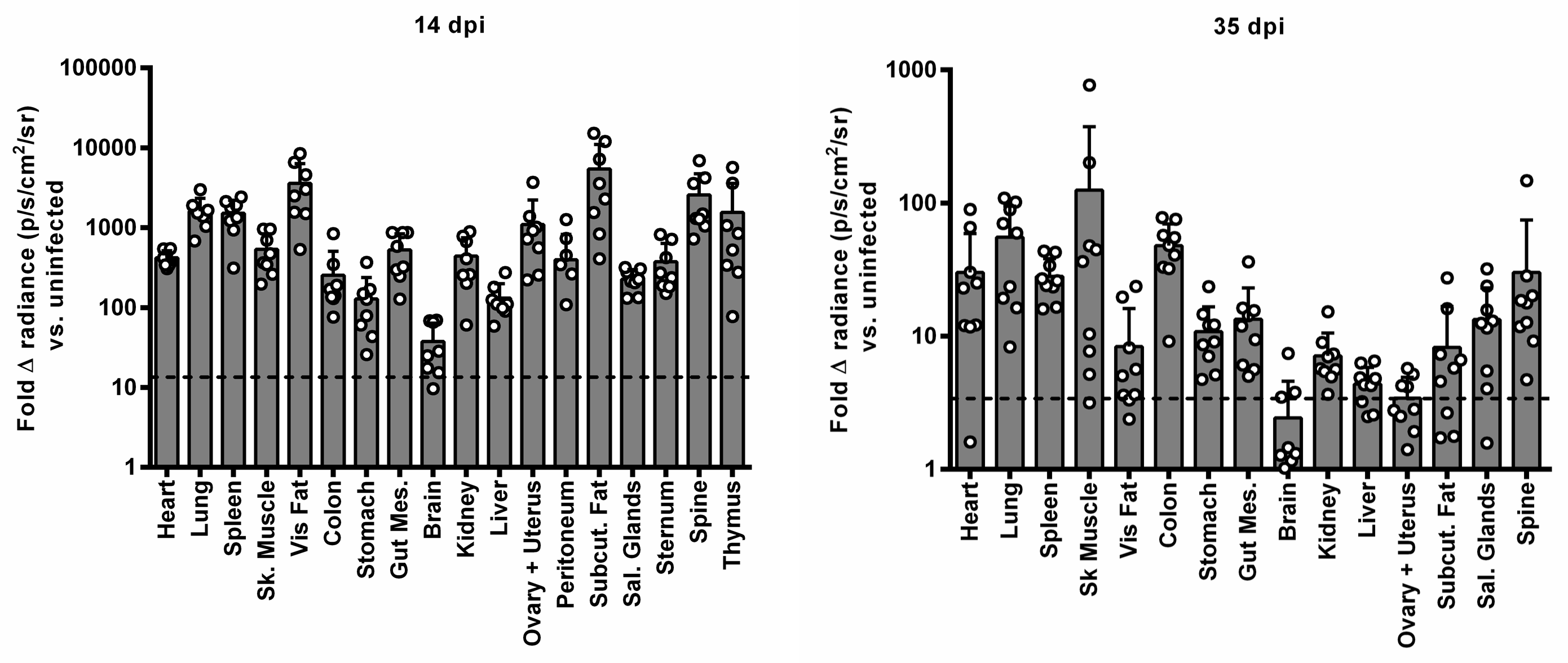

Supplement: Fig S4 — Tissue-specific parasite distributions and loads in acutely infected BALB/c mice. Quantification of ex vivo bioluminescence for selected organs and tissues taken immediately post-mortem from BALB/c mice infected with PpyRE9h luciferase-expressing T. cruzi. Animals were inoculated by i.p. injection of 1 × 10^3^ trypomastigotes and ex vivo imaging was performed at 14 dpi (n = 8, except lung n = 7 and peritoneum n = 6) and 35 dpi (n = 9 for all tissues). Data are means + SD of the fold-change in bioluminescence intensity for organs from infected mice compared with matching organs from uninfected mice and are pooled from two independent experiments (n ≥ 3 mice per time point per experiment). Dashed line indicates the detection threshold equal to the mean +2SDs of the fold-change in bioluminescence intensity for empty regions of interest (ROI) in the images obtained for infected mice compared with empty ROI in the images obtained for uninfected mice. [file cmi0016-1285-sd4.tif]
